# The Unexplored Importance of Fleeting Chiral Intermediates in Enzyme-Catalyzed Reactions

**DOI:** 10.1021/jacs.1c04551

**Published:** 2021-09-07

**Authors:** Manfred T. Reetz, Marc Garcia-Borràs

**Affiliations:** † Max-Planck-Institut für Kohlenforschung, Kaiser-Wilhelm-Platz 1, 45470 Muelheim, Germany; ‡ Tianjin Institute of Industrial Biotechnology, Chinese Academy of Sciences, 32 West 7th Avenue, Tianjin Airport Economic Area, Tianjin 300308, China; § Institute of Computational Chemistry and Catalysis (IQCC) and Departament de Química, Universitat de Girona, Carrer Maria Aurèlia Capmany 69, 17003 Girona, Spain

## Abstract

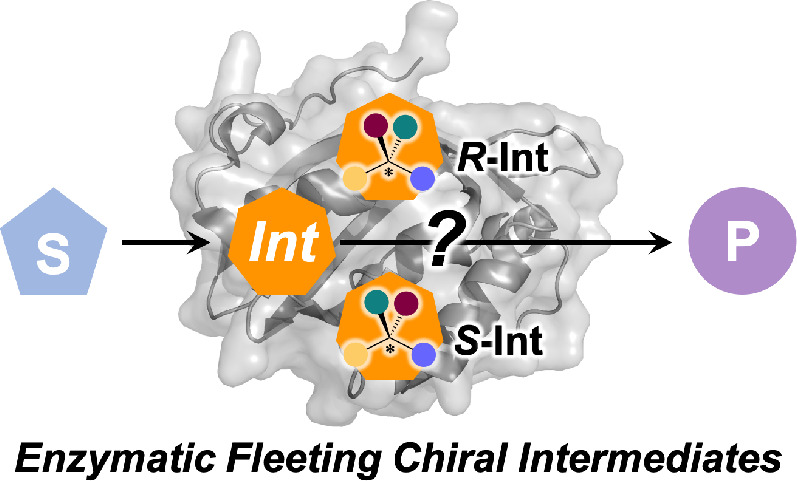

Decades of extensive research efforts by biochemists, organic chemists, and protein engineers have led to an understanding of the basic mechanisms of essentially all known types of enzymes, but in a formidable number of cases an essential aspect has been overlooked. The occurrence of short-lived chiral intermediates formed by symmetry-breaking of prochiral precursors in enzyme catalyzed reactions has been systematically neglected. We designate these elusive species as fleeting chiral intermediates and analyze such crucial questions as “Do such intermediates occur in homochiral form?” If so, what is the absolute configuration, and why did Nature choose that particular stereoisomeric form, even when the isolable final product may be achiral? Does the absolute configuration of a chiral product depend in any way on the absolute configuration of the fleeting chiral precursor? How does this affect the catalytic proficiency of the enzyme? *If these issues continue to be unexplored, then an understanding of the mechanisms of many enzyme types remains incomplete.* We have systematized the occurrence of these chiral intermediates according to their structures and enzyme types. This is followed by critical analyses of selected case studies and by final conclusions and perspectives. We hope that the fascinating concept of fleeting chiral intermediates will attract the attention of scientists, thereby opening an exciting new research field.

## 1 Introduction

Since life cannot be imagined in the absence of chiral molecules,^1,2^ scientists have wondered for decades about the origin of homochirality of amino acids in proteins and of nucleic acids in RNA and DNA.^2−8^ Apart from this continuing narrative, biochemists have invested great efforts in the quest to understand how enzymes catalyze selective reactions that lead to stable and isolable chiral compounds such as natural products or synthetic therapeutic drugs in enantiomerically pure or enriched form.^9−16^ Protein engineers have investigated the origin of improved or inverted stereoselectivity of evolved enzyme mutants by experimental and theoretical techniques.^9−13^ Unlike small organo- or metal-catalysts, where the chirality of the catalyst can be switched to obtain the opposite product stereoisomer, the scenario when enzymes are considered becomes much more complex. Prior to being released from the protein as products, these chiral molecules may exist in covalently or noncovalently enzyme-bound complexes, which sometimes could be “trapped” inside enzymes using spectroscopic or crystallographic techniques.^14−16^

Much less attention has been paid to a fundamentally different type of transient chiral intermediates which are formed over the course of many enzymatic reactions and which are unstable and nonisolable, elusively formed by breaking the symmetry of a prochiral precursor inside the protein. *We call them fleeting chiral intermediates, and pose the following key questions*: Does such an intermediate occur in homochiral form? If so, what is the absolute configuration, and why did nature choose that particular stereoisomeric form even when the final product may be achiral? If not, why not? Is this something one should consider when engineering enzymes to expand their substrate/reaction scope and catalytic proficiency?

*Without considering these issues, the complete understanding of the intricacies of many enzyme mechanisms remains unexplored*.

While fleeting chiral intermediates are usually formed in kinetically favored (fast) reaction steps and have short lifetimes, their formation and stabilization mode constitute a key to fully understanding catalytic performance in terms of activity and selectivity of natural and engineered enzymes. As will be seen in our Perspective, fleeting chiral intermediates are formed in different ways, depending upon the enzyme type and reaction mechanism.

**Scheme 1:**
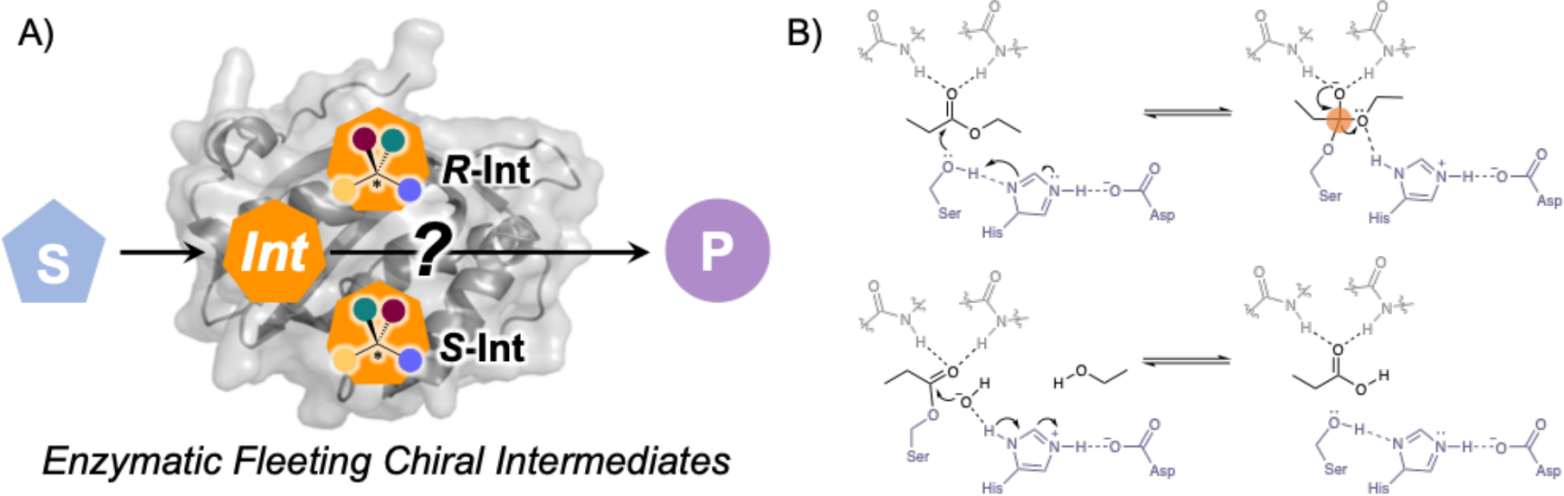
(A) Occurrence of fleeting chiral intermediates in enzyme catalyzed reactions (S: substrate; P: product; Int: fleeting chiral intermediate). (B) Standard mechanism of lipase-catalyzed hydrolysis, in this case of an achiral substrate (propionic acid ethyl ester) with formation of achiral products (ethanol and propionic acid), involving the formation of a chiral fleeting oxyanion (highlighted in orange) stabilized by backbone H-bonds originating from residues near the active site.

One wide realm concerns hydrolases such as lipases, esterases, or proteases,^17−22^ in which the activated hydroxy side chain of serine as part of the catalytic triad (Asp-His-Ser) adds nucleophilically to the carbonyl moiety of an acid, ester, lactone, or amide function with formation of a short-lived oxyanion (Scheme 1). The prochiral sp^2^ hybridized carbonyl C atom transforms into the fleeting chiral intermediate characterized by four different substituents at the newly formed sp^3^ hybridized C atom. The final products of interest need not be chiral themselves. Notice that, in Scheme 1B, a prototypical example of a lipase mechanism, the chiral oxyanion is not pictured as a three-dimensional intermediate, as it should be, a suboptimal convention that is adhered to in many textbooks, articles, and reviews.

Further structurally different fleeting chiral intermediates can be expected to occur in the mechanisms of different types of enzymes. A classification is therefore desirable that helps to identify them and to point the way for developing more complete mechanistic descriptions.

## 2 Proposed Classification of Structurally Different Fleeting Chiral Intermediates

Fleeting chiral intermediates with unique structures occur in very different enzyme families, involving different types of substrates and sometimes of cofactors. We propose the following classification:

### Cofactor-Free Enzymatic Processes

Among many possibilities, these include:

- Oxyanions with four different substituents at the respective C atom, occurring in the mechanism of lipases, esterases and proteases.^17−22^
- Protonated oxyanions with four different substituents at the tetrahedral C atom, formed in the multistep (retro)-aldolase reaction pathway from β-hydroxy ketones and aldehydes, but also in related enzyme catalyzed reactions that involve the formation of enamine and/or Schiff base intermediates covalently linked to catalytic lysine residues.^23−30^
- Oxyanions with four different substituents at the tetrahedral C atom as in racemases and epimerases.^31−33^
- Protonated oxyanions in which two of the four substituents at the respective tetrahedral C atom are formally identical, but are differently stabilized by supramolecular noncovalent H-bonding, as in the mechanisms of fluoroacetate dehalogenase,^34−37^ a carbonic anhydrase mutant,^38^ and most epoxide hydrolases.^39−42^

### Cofactor-Dependent Enzymatic Pathways

Among many others, these include:

- Oxyanions with four different substituents at the respective tetrahedral C atom, occurring in the mechanism of Baeyer–Villiger monooxygenases (BVMOs) involving a flavin-C4α-(hydro)peroxide reactive species and substrates containing ketone groups,^43−54^ as well as tetrahedral intermediates of other flavin-dependent monooxygenases, as in aromatic dehalogenation in which flavin-N5-peroxide adds nucleophilically to C(=O)–N moieties or C(Ar)–S bonds.^55−58^
- Short-lived chiral hydroperoxides occurring in the mechanisms of pyridoxal 5‘-phosphate (PLP) dependent decarboxylases and those involved in oxidative deamination^59,60^ and in related oxygen-dependent desaturation or hydroxylation found in enzymatic pathways.^61^
- Short-lived chiral geminal diamino species occurring in the mechanism of 5‘-phosphate (PLP) dependent transaminases and related enzymes.^62−68^
- Wheland intermediates formed during enzymatic electrophilic aromatic substitutions, as in flavin-dependent halogenases^69−72^ or in cofactor-free indole prenyltransferases that catalyze Friedel–Crafts alkylations of the indole aromatic ring.^73,74^

In the present Perspective, we focus on examples based on these enzymes, but note that other completely different kinds of fleeting chiral intermediates also occur in other enzyme mechanisms which likewise have not been analyzed with respect to the configuration of the reactive species. Additional examples also include short-lived chiral octahedral metal complexes, as in some enolases.^75−79^ Finally, short-lived chiral tetrahedral radicals undergoing fast and reversible racemization also need attention, as in the mechanism of P450-catalyzed oxidative hydroxylation^80−87^ and in other biological processes.^88−90^ The proposed classification should be continually extended to include additional enzymatic reactions in which fleeting chiral intermediates occur in already known enzyme catalyzed reactions, but also in newly discovered or artificially engineered enzymes.

Recent protein engineering efforts have led to the discovery of new abiological reactions that are catalyzed by natural and laboratory evolved enzymes. Some of these newly designed biocatalysts exploit the formation of short-lived chiral (radical) intermediates by utilizing unnatural substrate precursors and reaction conditions. These include, for example, carbene and nitrene transfer reactions catalyzed by Fe-heme dependent enzymes. Those involve the formation of octahedral iron-heme carbene^91,92^ and nitrene intermediates, being the first chiral when formed in the unsymmetric enzyme active site pocket, and which could induce the subsequent formation of C-centered fleeting chiral radicals via C–H activation.^93−95^ Other examples include the photobiocatalytic generation of enantioconvergent free radicals combining light with biological cofactors such are flavins or nicotinamides.^96−100^

These recent examples reinforce even more the importance of studying fleeting chiral intermediates during enzyme-catalyzed reactions. The complete understanding of their formation and their subsequent behavior is essential not only for deciphering the factors behind enzymatic efficiency and selectivity but also for designing new useful and selective biocatalysts for chemical synthesis.

## 3 Selected Case Studies

It is instructive to analyze a few case studies that are relevant to this Perspective. Rather than reviewing each selected publication with emphasis on the actual goal of the respective investigation, we focus critically on how the fleeting chiral intermediates were treated, if at all, and what the respective shortcomings mean in terms of understanding the intricacies of enzyme mechanisms.

### Lipases, Esterases, and Proteases

**Figure 1:**
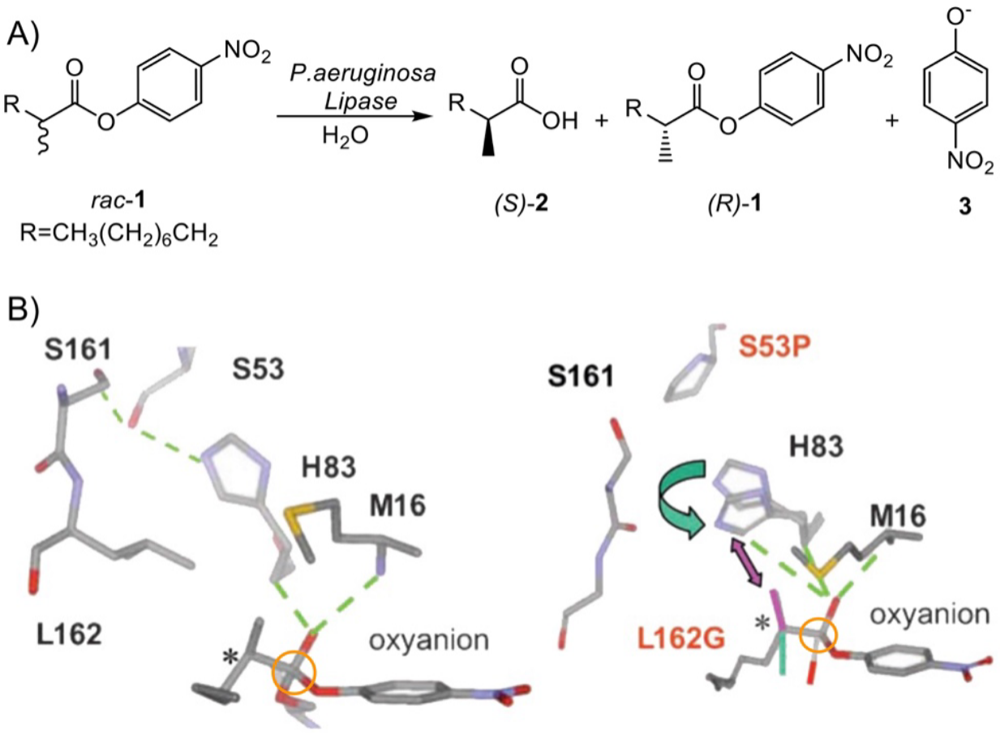
(A) Model kinetic resolution of substrate *rac*-**1** catalyzed by WT PAL and mutants with preferential formation of (*S*)-**2**.^22,101−103,105^ (B) On the left: Modeled oxyanion derived from substrate *rac*-**1** in WT PAL. On the right: Oxyanion derived from the favored substrate (*S*)-**1** (green, on the left) and the disfavored (*R*)-**1** (purple, on the right) in the double mutant S53P/L162G (*E* = 63); dotted green lines indicate H-bonds, asterisks indicate stereogenic centers. As shown by the crystal structure of PAL, the catalytic triad is H251-D229-S82.^104^ Figure adapted (B) with permission from ref (103). Copyright 2007 John Wiley and Sons.

In the field of directed evolution of stereoselective enzymes,^9−13^ no enzyme has been studied more systematically than the lipase from *Pseudomonas aeruginosa* (PAL), the hydrolytic kinetic resolution of 2-methyldecanoic acid *p*-nitrophenyl ester (*rac*-**1**) serving as the model reaction with preferential formation of (*S*)-**2** (Figure 1A).^9,22,101,102^ Wild-type (WT) PAL has a very low selectivity factor of *E* = 1.1, reflecting the relative rates of the two enantiomers, which was increased to *E* = 51 by applying a combination of random mutagenesis, focused saturation mutagenesis at residues lining the binding pocket, and DNA shuffling.^102^ This means that substrate (*S*)-**1** reacts 51 times faster than the enantiomer (*R*)-**1**. The evolved variant contains six mutations (D20N/S53P/S155M/L162G/T180I/T234S). In an initial study based on hybrid QM/MM calculations,^22^ the absolute configuration of the fleeting chiral oxyanion was not at all considered (Scheme 1B). In a second QM/MM study, it was predicted that most of the mutations introduced by random mutagenesis are superfluous, and that the double mutant S53P/L162G should be equally stereoselective or even better, which proved to be the case (*E* = 63).^103^ This PAL mutant was then computationally modeled using QM/MM calculations with the assumption of an absolute configuration of the oxyanion as shown in Figure 1B. This choice was made by crude inspection of the environment directly around the fleeting intermediate, and since qualitatively it seemed to fit better, this particular geometric arrangement was chosen. Computationally, both WT PAL and mutants were built from the available crystal structure (RCSB Protein Data Bank, ID 1EX9)^104^ complexed with the inhibitor *R*_c_-(*R*_P_,*S*_S_)-1,2-dioctylcarbamoyl-glycero-3-*O*-*p*-nitrophenyl octylphosphonate).^103^ The covalently bound tetrahedral inhibitor was then replaced by the oxyanion (Figure 1b). However, if a different inhibitor had been chosen, a different assumption concerning the configuration of the oxyanion may have been made, a clear weakness of this study. A direct structural and energetic comparison, if it had been made, would have provided new insights.

In a study of the kinetic resolution of acylated racemic tertiary alcohols catalyzed by lipase A from *Candida antarctica* (CALA),^106,107^ guided by previous works on phosphonate inhibitors,^108,109^ an absolute configuration of the oxyanion was chosen. However, the opposite configuration was not considered, which would have provided important and revealing information.

In a dissertation written at the Max-Planck-Institut für Kohlenforschung in 2006, some mechanistic aspects of the lipase from *Bacillus subtilis* (BSLA) and variants were studied using QM/MM calculations.^110^ This enzyme had been evolved in an earlier study^111^ for inverting the enantioselectivity in the hydrolytic kinetic resolution of *rac*-1-(2-naphthyl)-ethyl-acetate with formation of (*R*)- and (S)-1-(2-naphthyl)-ethanol.^110^ While WT BSLA is (*R*)-selective (*E* = 156), single mutant H76A resulted in inversion of enantiopreference in favor of the (*S*)-product(*E* = 8.5). Both configurations of the oxyanion were computed, with one of them being favored by about 2 kcal/mol. This in itself is an important advancement, but the respective *transition states* leading to each stereoisomeric product were not calculated.^110^ Thus, it is not clear whether the absolute configuration of the oxyanion correlates with the absolute configuration of the product.

Relevant is the well-known rule in asymmetric olefin hydrogenation, catalyzed by a man-made chiral Rh-catalyst, that the minor binding precomplex (5%) actually leads to the major product (95% ee).^112^ The fact that the distinctly better binding precomplex, identified by NMR spectroscopy, is not involved in the formation of the observed enantiomeric product, may appear odd, but the analogous question in enzymology should not be ignored.

The extensive literature of esterases reveals many important mechanistic details, but no information on the chirality of the respective oxyanions is explicitly discussed.^14,18,113,114^

The literature on the mechanism of proteases is even more vast, and the chirality of oxyanions has also been routinely ignored. Here, we mention only a single example case. In an intriguing recent study of the α-lytic protease, the question was posed: Why does lyophilization of this enzyme causes a structural change that is not reversed by redissolution in water?^115^ In their interpretation, the authors considered an oxyanion formed by nucleophilic addition of catalytically active S195 to the carbonyl C atom of the amide function in the polypeptide chain, and the crucial role of H57 in the formation of the oxyanion was proposed. However, the absolute configuration of the fleeting chiral oxyanion was not part of the analysis,^115,116^ and thus it is not clear if the chiral nature of this intermediate influences the observed irreversible structural change or not.

### Enzymes in Which the Fleeting Oxyanions Have Two Hydroxy Groups Stabilized Differently by Supramolecular Interactions in Active Sites

**Figure 2:**
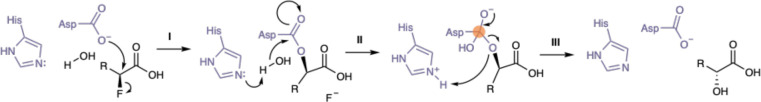
Example of fleeting chiral intermediate occurring in fluoroacetate dehalogenase. Mechanism of fluoroacetate dehalogenase when using a substituted chiral substrate.^34,35^ The formed fleeting chiral intermediate is highlighted in orange. Scheme redrawn based on ref (34).

Fluoroacetate dehalogenases (FAcD) catalyze the hydrolysis of toxic fluoroacetic acid with formation of glycolic acid and fluoride ions, and also play an essential role in the metabolism of organofluorine compounds.^117^ The mechanism as elucidated on the basis of the crystal structure of the FAcD RPA1163 from *Rhodopseudomonas palustris* CGA009 in the reaction of fluoroacetic acid involves classical S_N_2 substitution supported by the Asp-His-Asp triad.^36,37^ The question whether a Walden double substitution process with intermediate formation of an α-lactone is actually occurring, was recently answered by the use of a stereochemical probe employing substituted chiral substrates (Figure 2).^34,35^ Since inversion of configuration was observed, two successive inverting steps in a Walden process were discarded. His-activation of a water molecule enables nucleophilic addition to the carbonyl function of the intermediate ester with formation of a short-lived oxyanion (Figure 2), that would also be expected in the case of the achiral parent compound fluoroacetic acid. The situation is somewhat different from chiral oxyanions in lipases/esterases/proteases, since two of the four substituents are stabilized hydroxy groups, which means that on a superficial inspection, the intermediate is formally achiral. However, the hydroxy groups establish different H-bonds, thereby being supramolecularly stabilized in different ways due to specific interactions with different residues. In this sense, they are indeed chiral, with two different configurations being possible. A similar situation arises in the case of a mutant of carbonic anhydrase which acts as an esterase in a promiscuous reaction.^38^

**Figure 3:**
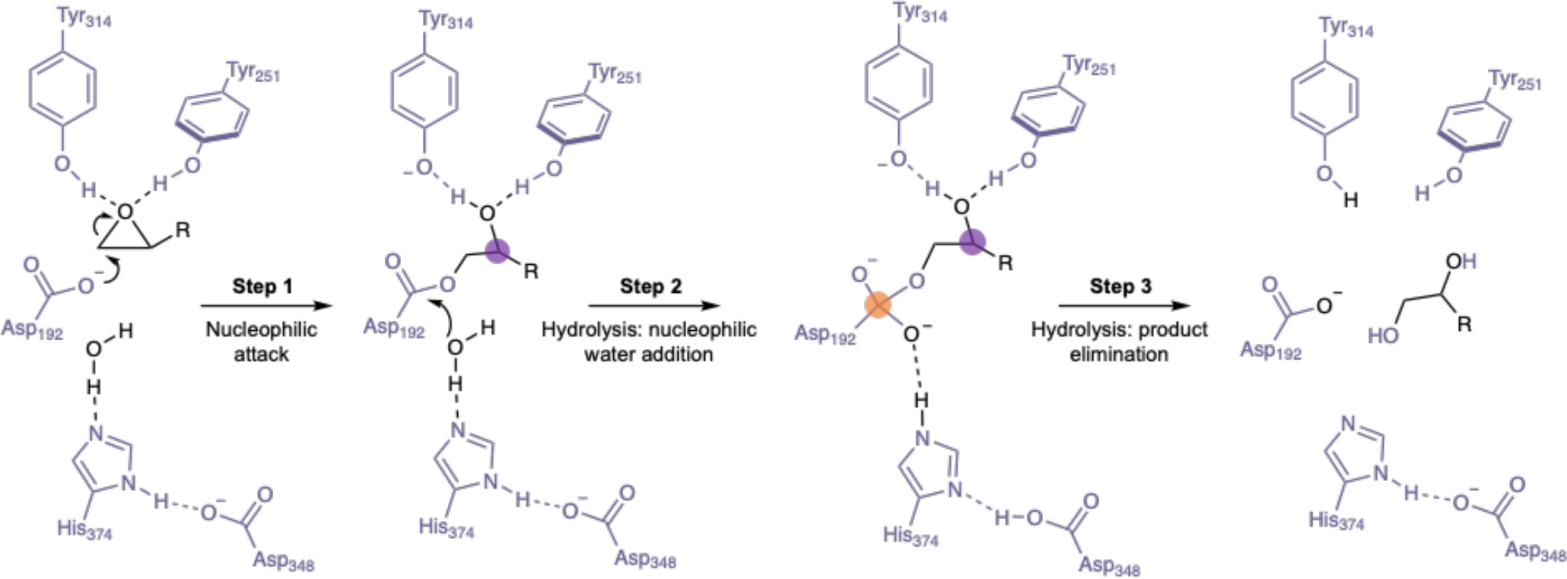
Example of fleeting chiral oxyanion intermediates occurring in epoxide hydrolase mechanism. Scheme of the *Aspergillus niger* epoxide hydrolase (ANEH) reaction mechanism involving the formation of a first chiral oxyanion (step 1) and a second fleeting chiral oxyanion (step 2, highlighted in orange) with two hydroxy groups that are differently stabilized by supramolecular H-bond interactions. Scheme drawn based on ref (39).

Further examples of this phenomenon occur in the mechanism of most epoxide hydrolases, including the one from *Aspergillus niger* (ANEH) in which rate-determining ring-opening by nucleophilic attack of an aspartate defines the general mechanism, followed by rapid hydrolysis.^39,40^ The latter step involves addition of a water molecule with formation of a fleeting oxyanion having two hydroxy groups at the central C atom, that are differently stabilized by supramolecular H-bond interactions by the enzyme’s asymmetric active site. This makes such an intermediate chiral. Unfortunately, the formation of this intermediate was not even considered in the original study. In fact, in the extensive literature covering epoxide hydrolases, it has never been highlighted to the best of our knowledge. This missing reaction step involving the formation of a fleeting chiral intermediate is shown in the new adapted scheme shown in Figure 3. It is possible that only one absolute configuration enables optimal stabilization due to diastereomeric interactions. A more in depth study based on extensive computational modeling with consideration of both configurations in an evolved enantioselective ANEH mutant^39^ and the WT^40^ would finally resolve this issue and lead for the first time to a complete mechanistic picture of epoxide hydrolases.

### Baeyer–Villiger Monooxygenases

Baeyer–Villiger monooxygenases (BVMOs) are flavin-dependent enzymes that occur in many different organisms and plants.^43,44,47−49,118^ Decades of intense efforts have uncovered the general features of the mechanism. Flavin is first reduced by NADPH, which then reacts with molecular oxygen (O_2_) to selectively form a C-alkylhydroperoxide at the C4α position.^43,44^ The C4α stereochemistry, as opposed to the alternative C4ß configuration, was chosen on the basis of early indirect experimental evidence, and has never been questioned.^44,47,48^ This intermediate then adds nucleophilically to the carbonyl function with formation of the tetrahedral Criegee-intermediate, which in the case of cyclohexanone is not chiral. However, if prochiral ketones such as 2-butanone are used as substrates, then the respective oxyanions are chiral, which has been largely overseen.

BVMOs have long been used in organic chemistry and biotechnology as catalysts in regio- and stereoselective transformations, often cyclohexanone monooxygenase (CHMO) from various sources serving as biocatalysts.^48,49^ When selectivity is poor or when reversal of regio- or enantioselectivity is desired, then directed evolution was successfully applied.^45,50−53^ Details of the mechanism were provided by a seminal computational study based on QM/MM calculations,^51^ aimed at elucidating the factors that lead to complete enantioselectivity in the desymmetrization of 4-methylcyclohexanone with formation of the respective (*S*)-lactone (95% ee) catalyzed by WT CHMO from the *Rhodococcus* sp. strain Hi-31. In the achiral Criegee intermediates originating from an energetically preferred orientation of unsubstituted cyclohexanone in the CHMO binding pocket, the potentially migrating C–C bonds are antiperiplanar to the peroxy bond, a stereoelectronic requirement for an energetically accessible migration. When using prochiral 4-methylcyclohexanone leading to the favored (*S*)-lactone, the oxyanion is also achiral, and the 4-methyl substituent is in the preferred equatorial position, while the reaction path to the (*R*)-lactone requires it to be axial. The energy difference between these two conformations was computed to be 2.3 kcal/mol.^51^ This theoretical study provided a viable model for subsequent BVMO investigations.^46,49,52,53^

**Figure 4:**
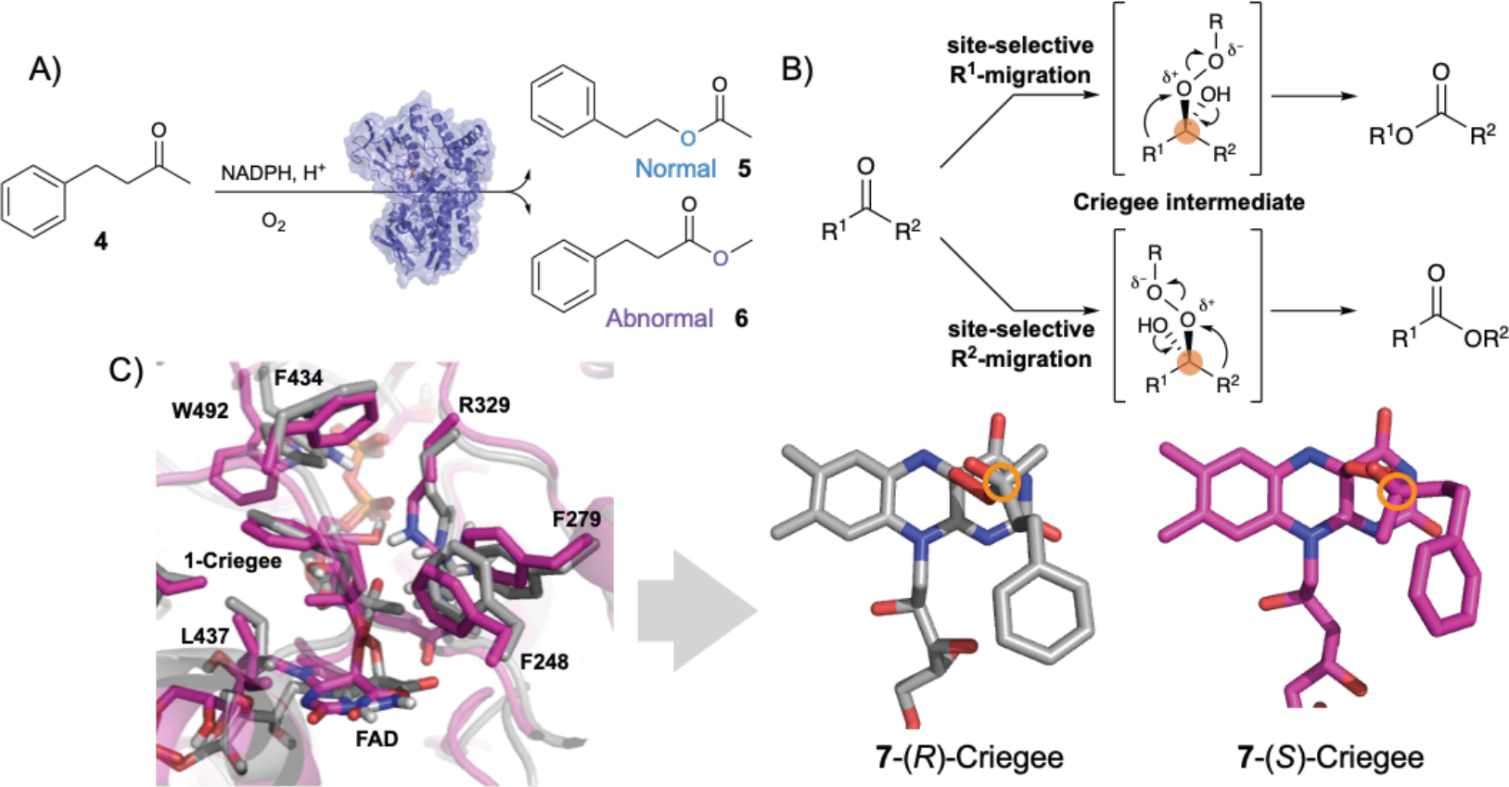
(A) Normal and abnormal reaction modes in the BVMO-catalyzed model reaction using 4-phenyl-2-butanone (**7**) as substrate.^53^ (B) Site-selective migrations lead to different products. (C) Overlay of representative snapshots obtained from MD simulations of **7**-*R*-Criegee (structure in gray) and **7**-*S*-Criegee (structure in pink) intermediates bound in WT T_m_CHMO.^53^ Figures redrawn (A, B) and adapted (C) with permission from ref (53). Copyright 2018 American Chemical Society.

One of the prime challenges in protein engineering of BVMOs is the reversal of regioselectivity from the generally preferred “normal” to the “abnormal” reaction mode.^45,48,49,53^ The best migrating groups are those that stabilize the partial positive charge best. The following migratory tendency has been established traditionally in organic chemistry in the absence of any enzymes: tert-Bu > Phe ∼ iso-Pr > Et > Me in organic chemistry without enzyme use. A protein engineering breakthrough was recently reported in which the technique of Combinatorial Active-site Saturation Test/Iterative Saturation Mutagenesis (CAST/ISM)^9,119^ was applied to the BVMO from *Thermocrispum municipale* DSM 44069 (T_m_CHMO) as the catalyst in the reaction of 4-phenyl-2-butanone (**4**) (Figure 4A).^53^ While WT T_m_CHMO favors the expected migration of the 2-phenylethyl group with formation of the normal product **5** (99:1), quadruple mutant LGY3-D-E1 (L145G/F434G/T435F/L437T) reverses regioselectivity completely in favor of the abnormal product **6** (2:98).^53^

In order to explain this dramatic switch, docking calculations, extensive MD simulations, and QM/MM computations were performed.^53^ These showed that mutations introduced by directed evolution cause crucial changes in the conformations of the respective Criegee intermediates and transition states (Figure 4B). It was found that in the case of the abnormal-selective mutant LGY3-D-E1, the respective mutations destabilize the migration transition state of the normal reaction pathway, rather than favoring the activity of the abnormal reaction. Such a conformational control ensures ideal O–O–C–C dihedral angles and overrides electronic control which usually ensures preferential migration of the group that stabilizes the incipient positive charge at the peroxy-oxygen atom best. The (*R*)-configurated Criegee intermediate in WT T_m_CHMO and in the LGY3-D-E1 mutant was used in the mechanistic QM/MM modeling. This choice was based on extensive MD simulations in which both the (*R*)- and (*S*)-Criegee intermediates were explicitly modeled when formed in the binding pocket of WT T_m_CHMO. These showed that the formation of the (*S*)-enantiomer is not likely to occur due to the strained geometry it would have as compared to the corresponding (*R*)-Criegee intermediate (Figure 4C). However, it is unfortunate that the energetic pathway involving the alternative Criegee enantiomer was not computed, which would have provided interesting mechanistic insights.

Other flavin-dependent monooxygenases follow similar mechanisms which likewise involve covalent tetrahedral intermediates. These include, for instance, recently characterized flavoenzymes that employ flavin-*N*5-peroxide as nucleophiles for catalysis, instead of C4-peroxide. These enzymes are found to catalyze the redox-neutral cleavage of carbon-heteroatom bonds (C(=O)–N, and C(ar)–S) and aromatic dehalogenation via nucleophilic oxygenation.^55−58^ Recent efforts to structurally characterize the regiospecific functionalization of the flavin cofactor in the enzyme active site and how these affect the subsequent covalent intermediates along the reaction pathways provide a basis for further computations and laboratory experiments,^55,120^ which will hopefully explain the chirality of the respective fleeting intermediates.

Additionally, other enzymatic oxidation reactions may also involve the formation of fleeting covalent intermediates, as for example alkene epoxidations catalyzed by Fe-heme P450s and peroxygenases that use Compound I (Cpd I, Fe=O) as the oxidative species, and that are shown to also form covalent tetrahedral transient intermediates.^121,122^ When prochiral alkenes are used, such fleeting intermediates may well be chiral.

### Fleeting Chiral Wheland Intermediates in Enzymatic Reactions

Wheland intermediates are arenium ion sigma-complexes that in synthetic organic chemistry occur in electrophilic aromatic substitution (S_E_Ar) reactions.^123−125^ In the case where unsymmetric or substituted aromatic rings are involved, the corresponding Wheland intermediates are chiral. There exist a variety of enzyme-catalyzed electrophilic aromatic substitution reactions, with the assistance or not of cofactors, that involve the formation of chiral Wheland intermediates. For example, in FAD-dependent halogenases,^69−72^ or in Friedel–Crafts alkylation reactions catalyzed by indole prenyltransferases.^73,74^

**Figure 5:**
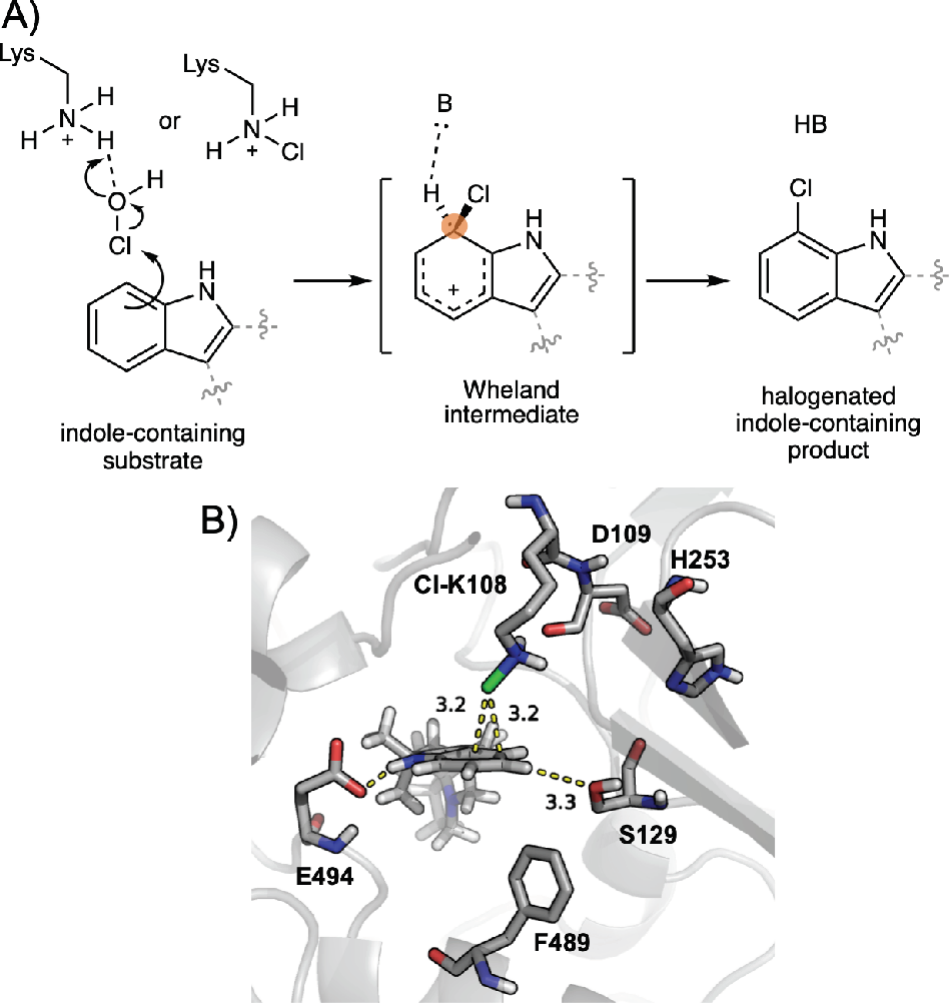
Example of a fleeting chiral Wheland intermediate formed over the course of FAD-dependent halogenases catalyzed reactions. (A) Mechanism of chlorination of indole-containing substrates by FAD-dependent halogenases, which follows a two-step aromatic substitution reaction (S_E_AR): a first reaction step forms a Wheland intermediate, and a second deprotonation step leads to the final halogenated product. (B) Representative snapshot obtained from MD simulations of MalA′ and premalbrancheamide substrate-bound complex, including the chloramine adduct at K108 (Cl–K108) and potential deprotonating residue S129. Figure adapted with permission (B) from ref (71). Copyright 2017 American Chemical Society.

In the particular case of flavin-dependent halogenases, elusive chiral Wheland intermediates have been proposed to occur over the course of the enzymatic reaction. FAD-dependent halogenases catalyze the chlorination of aromatic substrates, such as tryptophan or indole derivatives, following a two-step aromatic electrophilic substitution (S_E_Ar): First insertion of a chlorine atom forming a Wheland intermediate, and final deprotonation of the arenium ion intermediate that rearomatizes the system and leads to the final product (Figure 5).

These enzymes utilize FADH_2_ to reduce O_2_ to water while oxidizing a chlorine ion to hypochlorous acid (HOCl) that takes place in the FAD binding site and which is different from the substrate binding pocket. Depending on the enzyme structure, this HOCl species is proposed to migrate to the substrate binding site through a tunnel in the enzyme and become electrophilically activated by an H-bond interaction with a catalytic lysine nearby the substrate. It is proposed that HOCl could directly be the chlorinating species^70^ or that it could form a long-lived lysine chloramine species that will be responsible to transferring the chlorine to the substrate.^126^ Irrespective of the question of which of these species, HOCl or lysine chloramine, act as the final chlorinating agent, the formation of a fleeting Wheland intermediate during the first step of the S_E_Ar reaction was proposed and indeed it has been computationally shown to be possible.^71,72^ These computational studies have analyzed the mechanism of FAD-dependent halogenases, focusing on the selectivity of the chlorination step involving substrates that include an indole ring in their structures (such as indole alkaloids or tryptophan).

Tryptophan 7-halogenase PrnA catalyzes the selective chlorination of tryptophan at the C7 position,^69^ which corresponds to the first step in the biosynthesis of pyrrolnitrin and rebeccamycin antibiotics. A computational work based on QM/MM hybrid calculations studied the formation of the Wheland intermediate during the first step of the S_E_Ar reaction, considering HOCl as the chlorinating agent.^72^ Based on the binding pose observed for the 7-chlorotryptophan in the available product-bound X-ray structure (PDB: 2AR8), and how the HOCl···K79 catalytic species should approach the C7-indole position, the chirality of the Wheland intermediate was assumed. The proposed stereoisomer for the Wheland intermediate could then be easily deprotonated by the E346 active site residue, which is also H-bonding with the protonated N-amine group of the indole ring. Rearomatizing the 6-membered ring leads to the final product.

In a different work based on the newly characterized FAD-dependent halogenase MalA,^71^ the formation of a fleeting Wheland intermediate was also computationally modeled. Ma1A halogenase performs iterative late-stage halogenation of complex substrates independent of a carrier protein, and it is involved in the biosynthesis of malbrancheamide indole alkaloid.^127^ It catalyzes the chlorination of the premalbrancheamide substrate at the C8 and C9 positions of the indole ring. The available substrate-bound X-ray structure (PDB: 5WGR) was used as a starting point for computational modeling using MD simulations. These highlighted the arrangement of active site waters and an active site S129 residue, which can interact with H–C9 and H–C8, respectively, when the substrate is in an appropriate orientation for the electrophilic aromatic substitution considering a lysine chloramine as the chlorinating species. DFT calculations, based on computational truncated models, demonstrated that a water molecule or a serine side chain interacting with the C8/C9 protons enhances the chlorination by increasing the nucleophilicity of these carbons when forming the corresponding Wheland intermediates. According to the DFT calculations, the final deprotonation step leading to the rearomatization of the indole ring and forming the final product, appears to be smooth due to the preorganized water molecule or serine side chain. Mutagenesis experiments corroborated the importance of S129 to activate C8 position for chlorination.

These studies indicate that there is only one reactive binding pose of the substrate and that the chlorinating species can only approach the substrate from one π-face. Consequently, the Wheland intermediates are stereospecifically formed during the S_E_Ar in these halogenase reactions. Polar residues occur specifically in the enzyme active sites to establish key polar interactions that activate the substrates and enable the final deprotonation of the arenium ions. These results suggest that the chirality of the fleeting Wheland intermediates formed in these two different FAD-dependent halogenases is unique and that it plays a key role in the proficiency of the enzyme, although the final products are not chiral. However, this has not been explicitly highlighted in the published studies.

### Fleeting Chiral Intermediates in Pyridoxal 5‘-Phosphate Dependent Enzymes

Pyridoxal 5′-phosphate (PLP) is the bioactive form of vitamin B_6_, which acts as a cofactor in a large variety of important enzymatic reactions such as transaminations or many reactions involving natural amino acids (decarboxylation, deamination or racemization reactions).^128^

One characteristic of PLP is that its aldehyde group can form an internal aldimine (a Schiff base) which is covalently attached to the amino group of a catalytic lysine in the enzyme active site. This Schiff base can then further react with the amino group of the amino acid substrate to form an external aldimine, which subsequently undergoes different transformations depending on the enzyme (deprotonation, loss of CO_2_, forming a quinonoid intermediate, etc.). Due to their versatility, PLP-dependent enzymes have become a very important family of enzymes in biocatalysis.^59,60,62−68,128^

In particular, transaminases have emerged as an important class of enzymes that catalyze the reductive amination of prochiral ketones with formation of pharmaceutically highly sought-after chiral amines.^62−68,129^ Directed evolution has been applied to expand their substrate scope, and to enhance or inverting enantioselectivity for useful synthetic purposes.^63,66,68^ Synthetically relevant chiral amines can also be obtained from other biocatalytic routes, for instance via deracemization of mixtures of chiral amines using chemo-enzymatic methods involving FAD-dependent monoamine oxidases (MAO). Laboratory evolved MAO-N variants can catalyze the enantioselective oxidation of the nondesired amine enantiomer to afford the corresponding imine or iminium ion, which is then chemically reduced to the racemic starting material, thus accumulating the desired amine enantiomer after several rounds of oxidation and reduction in a highly efficient manner.^130−133^

**Figure 6:**
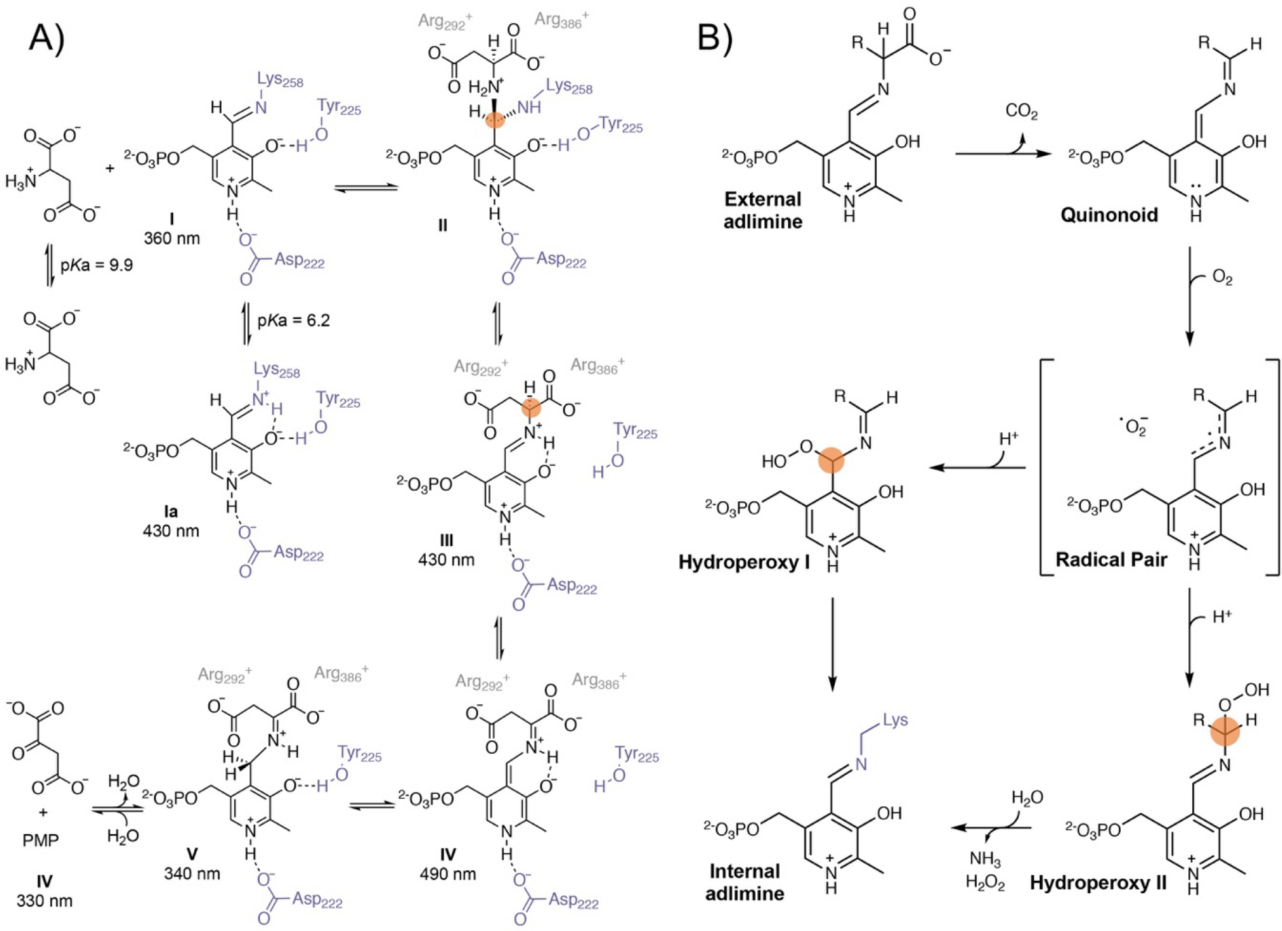
(A) Mechanism of transaminases explicitly featuring the transamination step that involves the formation of a chiral geminal-diamino intermediate (upper right). Redrawn from ref (134). (B). Proposed mechanism for oxidative deamination catalyzed by pyridoxal 5‘-phosphate dependent decarboxylases. Redrawn from ref (60).

The PLP-dependent transaminase’s reaction mechanism was postulated as early as 1984, according to which the formation of short-lived germinal diamino intermediates was noted^134^ (Figure 6A). Their formation was confirmed by X-ray crystallography in a related ornithine decarboxylase PLP-dependent enzyme variant having two mutations.^135^ However, since the two introduced mutations were found to affect and modify the enzyme active site, it is not clear if the captured geminal-diamine intermediate corresponds to the active enantiomer on the reaction path, or to an inhibited dead end pathway. Computational studies on this system were carried out based on the use of truncated models of the enzyme active site. Calculations considered only the pathway involving the X-ray characterized diamino intermediate, and concluded that the rate-limiting step of the transimination reaction corresponds to the subsequent proton transfer step.^136^ It could be that this particular enantiomer may be less reactive in the proton transfer step than the other enantiomer which was not trapped in the X-ray structure. Since this alternative reaction pathway was not computed, the role of chirality remains uncertain.

A small number of pyridoxyl 5′phosphate dependent enzymes employ molecular oxygen as a cosubstrate, which expands even more the catalytic toolbox of this family of enzymes. Although it is not fully clear how the PLP cofactor and O_2_ react, two different activating modes have been proposed. Analogous to flavoproteins, it has been suggested that from the quinonoid intermediate a single electron transfer to O_2_ leads to a radical pair which can undergo two different subsequent reactions with formation of two different short-lived chiral intermediates (hydroperoxyl I and II, Figure 6B).^59,60,137,138^ It is also theoretically possible that the quinonoid could be activated by excitation to its triplet state and then reacts with the triplet O_2_ molecule, generating the corresponding hydroperoxyl intermediates.

The formation of these highly reactive hydroperoxides, independently of how they are generated, correspond to fleeting chiral intermediates that could be differently stabilized depending on the enzymes and substrates involved. Consequently, in order to completely characterize these intriguing biocatalytic pathways, it is essential to identify the specific chirality of these fleeting intermediates.^60^

## 4 Conclusions and Future Perspectives

Fleeting chiral intermediates, as we have defined them here, occur in a multitude of enzyme-catalyzed reactions, but consideration of their absolute chirality has been routinely neglected, sometimes because the final products are achiral or because they are not believed to play an important role in the enzymatic mechanism. These include important enzyme families that catalyze common biocatalytic reactions of synthetic interest or important biocatalytic routes in the biosynthesis of natural amino acids and natural products (proteases, lipases and esterases, flavin-dependent halogenases, monooxgenases, PLP-dependent enzymes, etc.) as reviewed in the above sections. In some particular cases, as in flavin-dependent halogenases, the chirality of the fleeting chiral intermediates (a Wheland intermediate) was believed to occur in Nature, although not explicitly highlighted as such. Based on structural and mechanistically insights, it was assumed that the fleeting Wheland intermediates in the studied FAD-dependent halogenases occur in a homochiral form, and that this is important to establish key interactions with active site residues that are involved in activating the substrate and in the final deprotonation step of the reaction, controlling the regioselectivity of the whole process. For these enzymes, a very good mechanistic understanding has been developed. In principle, full characterization of enzyme mechanism helps to rationally engineer new mutants with alternative selectivities.^71,127,139,140^ In the case of most of the enzyme types that we have highlighted here, this still has to be achieved.

The occurrence of fleeting chiral intermediates is not limited to natural enzymatic reactions. Examples are a new family of abiological carbene and nitrene transferases (mentioned above) or *artificial* carboligases. The latter enzyme family, that arose from a *de novo* computationally designed retro-aldolase followed by extensive laboratory evolution, catalyzes multistep reactions utilizing covalently linked enamines and Schiff base intermediates that are formed from chiral intermediate species.^12,23−26,141−145^ The formation of these chiral intermediate species was not considered at all during the initial rational design procedure, and thus, the enzyme active site was not optimized to properly stabilize them. This could also be one of the reasons why *de novo* enzymes often perform less efficiently as compared to Nature and laboratory evolved variants.^23,144,145^

In summary, we have pointed out numerous intriguing examples of different kinds of fleeting chiral intermediates that occur in many different enzymatic systems, with and without the participation of cofactors. The transformations are not only of biosynthetic interest. They are also involved in the regulation of metabolism in living systems. The question of why Nature chose a particular chirality for a given fleeting intermediate in a naturally occurring transformation, which thus far has not been conclusively identified, needs to be addressed more closely in future work. Does the absolute chirality of such an intermediate really matter for the proper function of that particular enzyme? The same uncertainties need to be resolved when attempting to understand the precise role of mutational results of directed enzyme evolution studies and when aiming to (re)design new enzyme variants. New and more efficient experimental and computational protocols would emerge. We hope that the fascinating concept of fleeting chiral intermediates will attract the attention of scientists and thereby open an exciting new research field. It will ultimately allow a full understanding of enzymatic catalysis, thereby expanding the applications of biocatalysis and positively influencing protein engineering and drug design.
